# Autophagosome formation in relation to the endoplasmic reticulum

**DOI:** 10.1186/s12929-020-00691-6

**Published:** 2020-10-22

**Authors:** Yo-hei Yamamoto, Takeshi Noda

**Affiliations:** Center for Frontier Oral Sciences, Graduate School of Dentistry, Osaka University Graduate School, 1-8 Yamadaoka, Suita, Osaka 565-0871 Japan

**Keywords:** Autophagy, Autophagosome, Endoplasmic reticulum, ERdj8/DNAJC16, Atg2, VMP1, TMEM41b, ATG9, COPII, PIS, CDIPT

## Abstract

Autophagy is a process in which a myriad membrane structures called autophagosomes are formed de novo in a single cell, which deliver the engulfed substrates into lysosomes for degradation. The size of the autophagosomes is relatively uniform in non-selective autophagy and variable in selective autophagy. It has been recently established that autophagosome formation occurs near the endoplasmic reticulum (ER). In this review, we have discussed recent advances in the relationship between autophagosome formation and endoplasmic reticulum. Autophagosome formation occurs near the ER subdomain enriched with phospholipid synthesizing enzymes like phosphatidylinositol synthase (PIS)/CDP-diacylglycerol-inositol 3-phosphatidyltransferase (CDIPT) and choline/ethanolamine phosphotransferase 1 (CEPT1). Autophagy-related protein 2 (Atg2), which is involved in autophagosome formation has a lipid transfer capacity and is proposed to directly transfer the lipid molecules from the ER to form autophagosomes. Vacuole membrane protein 1 (VMP1) and transmembrane protein 41b (TMEM41b) are ER membrane proteins that are associated with the formation of the subdomain. Recently, we have reported that an uncharacterized ER membrane protein possessing the DNAJ domain, called ERdj8/DNAJC16, is associated with the regulation of the size of autophagosomes. The localization of ERdj8/DNAJC16 partially overlaps with the PIS-enriched ER subdomain, thereby implying its association with autophagosome size determination.

## Background

Autophagy (specifically macroautophagy) is an intracellular degradation system that maintains cellular homeostasis and is associated with many pathophysiological phenomena, including cancer and neurodegenerative diseases [1, 2]. Autophagy involves membrane structures called autophagosomes, which envelop the substrates and subsequently fuse with the lysosomes, resulting in the degradation of the substrates [3]. In the last 30 years, the molecular mechanism of autophagosome formation has been progressively understood. Autophagosome formation is controlled by a group of proteins called Atg proteins, which are evolutionarily conserved in eukaryotes [4, 5].

There are two types of autophagy: non-selective and selective. Non-selective autophagy engulfs cytoplasmic solution non-selectively, which includes soluble proteins among others. The diameter of non-selective autophagosomes is thought to be relatively uniform (~ 1 μm). Selective autophagy engulfs diverse substrates of various sizes while large autophagosomes enwrap large autophagic substrates such as mitochondria and bacteria [6–9]. The size of autophagosomes varies from a few hundred nanometers in diameter to over a micrometer [10, 11]. Recently, we have reported that the ER resident membrane protein, ERdj8/DNAJC16, is involved in the size determination of autophagosomes [12]. Herein, we introduce recent advances in the relationship between autophagosome formation and endoplasmic reticulum.

## Main text

The endoplasmic reticulum (ER) is an organelle that plays diverse roles, such as the synthesis of secreted proteins and membrane proteins [13], protein transport [14], misfolded protein degradation [15], lipid synthesis [16], and calcium storage [17].

**Fig. 1:**
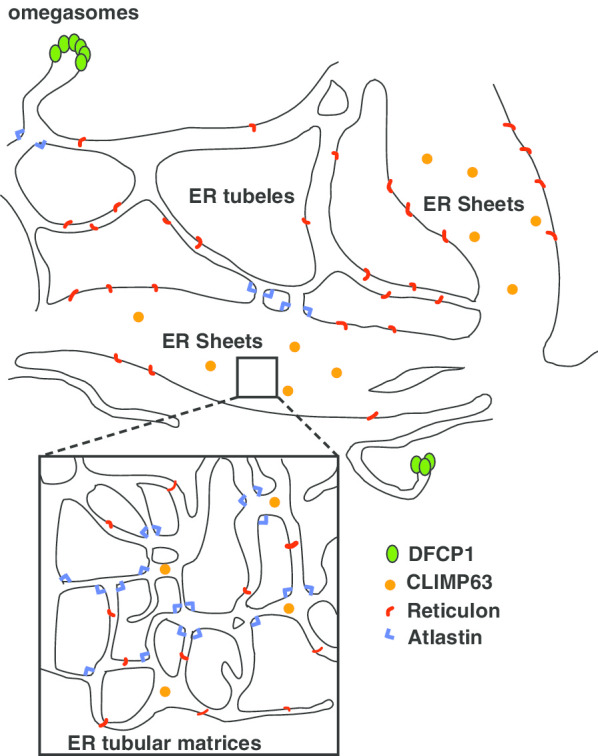
Schematic representation of the endoplasmic reticulum (ER) and the ER subdomain. The ER network is composed of tubules and sheet-like structures that are formed by the ER-shaping proteins, of which reticulon (red) creates the curvature of the ER membrane. CLIMP-63 (orange), which is a single spanning ER membrane protein, forms an oligomer into the ER luminal space to maintain the ER sheet structure. Atlastin (blue) localizes on the three-way junction of the ER network to create an interconnection of the ER tubules. The inset region shows the ER tubular matrices within the ER sheet structures. DFCP1 (green) localizes and forms the omegasome structures on the ER subdomain

The architecture of the ER is composed of a tubular network and sheet like structures; recent advances in super-resolution microscopy imaging techniques have revealed that the ER sheet structure consists of a dense assembly of ER tube structures [18] (Fig. 1). The tubular structures are maintained by a family of proteins termed reticulons [19, 20]. Cytoskeleton-linking membrane protein 63 (CLIMP-63) is responsible for the maintenance of the sheet-like structures [21]. The tubular structures are maintained by constant membrane fission and fusion, while the GTPase Atlastin regulates ER membrane fusion [22, 23]. Additionally, a type of subdomain within the ER, was recently identified; for example, a subset of ER membrane proteins such as Sec61b and Atlastin localize to specific regions within the ER [18].

The intracellular site where autophagosome formation takes place had earlier been the subject of controversy; however, it is now clear that it occurs near the ER subdomain called the omegasome [24] (Fig. 1). Double FYVE domain-containing protein (DFCP1) is a phosphatidylinositol 3-phsosphate binding protein that serves as a specific marker of omegasome [24]. Indeed, local turnover of phosphatidylinositol 3-phsosphate occurs in the omegasome by the balanced activity of class 3 phosphatidyl inositol 3-kinase complex containing Atg14L and phosphatase, such as MTMR3 [25, 26]. Ultrastructural analyses have revealed that autophagosomes emerge from the vicinity of the ER [27–29]. Further, autophagosome formation in yeast takes place near a specific region of the ER (ER exit sites) [30, 31] and the nuclear membrane [32]. The ULK1 complex, a scaffold of Atg proteins, is localized in the tubular vesicular ER subdomain where autophagosome formation occurs [33].

Recent advances have helped in understanding the relation between ER and autophagosome formation. Several reports have demonstrated the critical role of Atg2 in the connection between ER and autophagosomes both in mammalian and yeast cells [34, 35]. Atg2 binds to the ER along with Atg9 and Atg18 to form autophagosomes at the contact site between the autophagosome and the ER subdomain [34, 36]. A part of Atg2 shows homology to Vps13, which transfers lipid molecules from one organelle to another at the contact site [37]. Indeed, Atg2 shows lipid transfer activity, suggesting that lipid molecules flow from the ER to the autophagosome via Atg2 protein [35, 38, 39].

The lipid molecules that flow into the autophagosomes may not be preexisting ER constituents. Phospholipid synthases like phosphatidylinositol synthase (PIS)/CDP-diacylglycerol-inositol 3-phosphatidyltransferase (CDIPT) and choline/ethanolamine phosphotransferase 1 (CEPT1), accumulate in the ER subdomain and promote local lipid synthesis and elongation of the tube structure [40]. The small GTPase Rab10 is involved in the organization of the ER subdomain [40]. It is noteworthy that autophagosome formation takes place near this ER subdomain; the localization of the ULK1 complex occurs in the proximity of the PIS-enriched region [41]. Indeed, de novo phosphatidylcholine synthesis is required for autophagosome formation [42]. In yeast, de novo synthesis of fatty acids at the autophagosome formation site is further reported to be essential [43]. Therefore, it is possible that phospholipids synthesized near the autophagosome formation site are utilized. VMP1 is a multiple transmembrane ER protein [44–47]. Ablation of VMP1 inhibits the formation of PIS and CEPT1 subdomains of the ER [46] and leads to accumulation of the ULK1 complex and other autophagic machinery at the ER isolation membrane contact [47]. Another ER membrane protein called TMEM41b is also presumed to collaborate with VMP1 in these processes [48, 49]. Thus, these proteins play pivotal roles in the ER subdomain involved in autophagosome formation.

Are these Atg2-mediated lipid transfers the sole lipid source of autophagosome membrane formation? Coat protein complex II (COPII) vesicles, which bud from the ER, are also suggested to be involved in autophagosome formation [50]. Atg9 is a transmembrane protein that localizes to the autophagosomal membrane and transports vesicles between the Golgi and endosomes [51]. The Atg9 vesicle, which is a transport vesicle in which the Atg9 protein resides, is further suggested to constitute a part of the autophagosome [52], although the other possibility is also discussed [51]. The involvement of recycling endosome derived vesicles has also been reported [53]. Future studies are needed to understand the full repertoire of the lipid source of the autophagosome membrane.

**Fig. 2:**
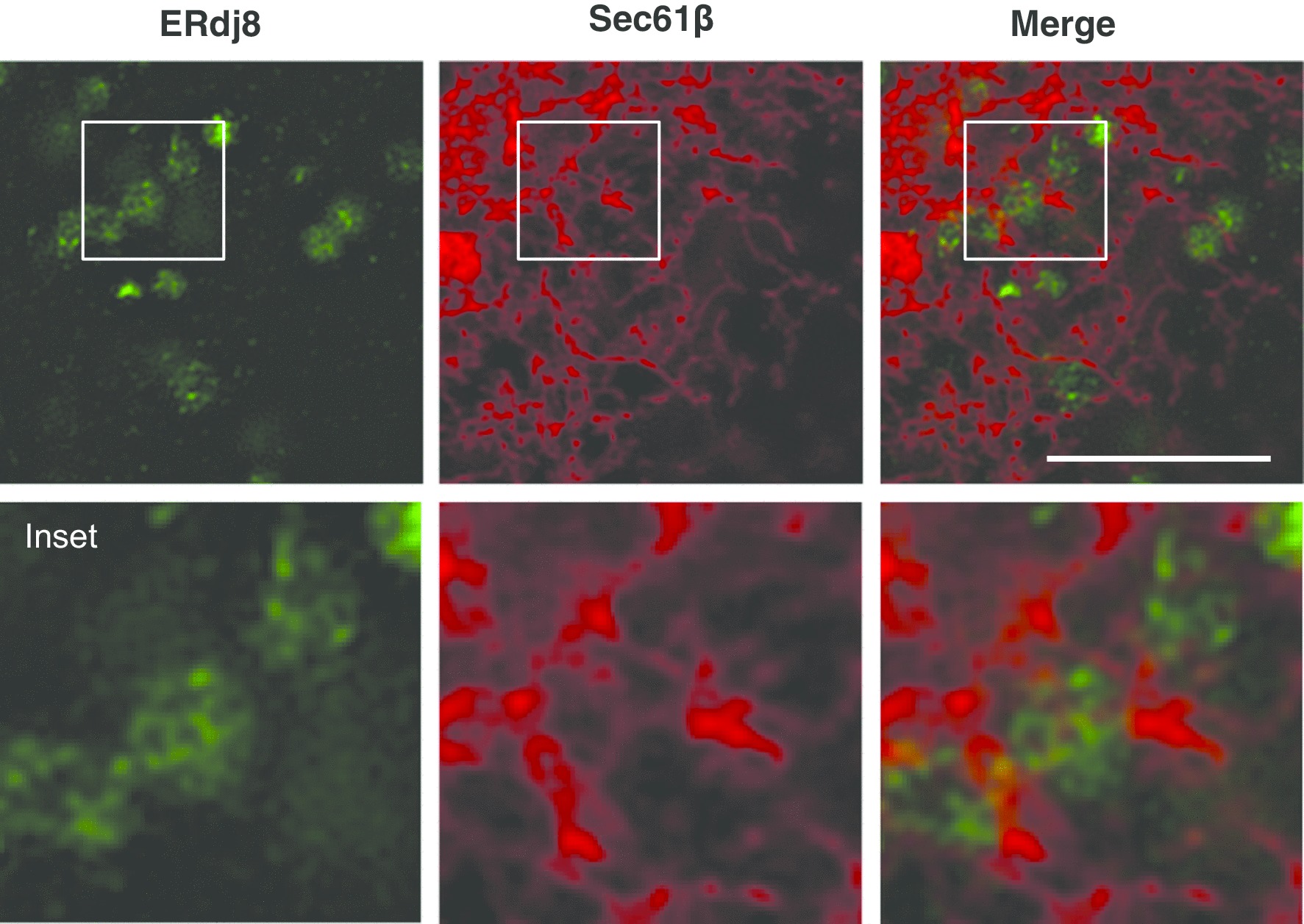
ERdj8 localizes at the ER subdomain. Cos7 cells expressing RFP-Sec61β were stained with anti-Erdj8 and imaged by super-resolution microscopy (SpinSR10, OLYMPUS). Scale bar is 10 μm. ERdj8 forms the ER meshwork structures near the Sec61β weak signal and creates the ER subdomain

**Fig. 3:**
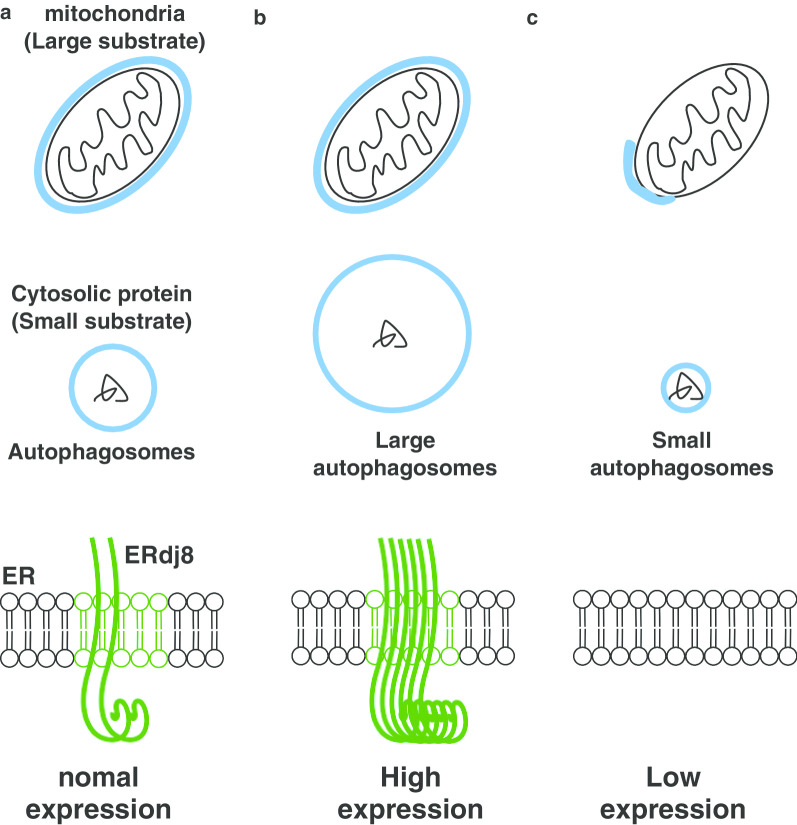
The expression level of ERdj8 alters the size of the autophagosomes. **a** Autophagosomes can capture both small and large substrates when ERdj8 expression is at the endogenous level. **b** ERdj8 overexpression induces the expansion of autophagosome membrane and both small and large substrates are degraded by autophagy. **c** Knockdown of ERdj8 induces the small autophagosomes to capture small substrates, whereas the degradation of large substrates is disabled

The ERdj proteins are a family of proteins harboring the DNAJ domain that cooperate with the molecular chaperones and play diverse roles in the ER [54–56]. Recently, we analyzed their uncharacterized member, ERdj8 [12]. ERdj8 was partially colocalized with PIS-enriched ER subdomains and also partially colocalized with Atg proteins (Fig. 2). When ERdj8 was ectopically overexpressed, there was an increase in the diameter of the autophagosomes in non-selective autophagy. When the expression of ERdj8 was knocked down, the size of nonselective autophagosomes was reduced. Moreover, knockdown or knockout of ERdj8 resulted in the failure of autophagosomes to enwrap large substrates such as mitochondria and latex beads with a diameter of 3 μm, while engulfment of smaller substrates such as paternally derived mitochondria or latex beads with a diameter of 1 μm was not affected (Fig. 3. Based on these results, we propose that ERdj8 regulates the size of the autophagosomes. The underlying mechanism remains to be determined; however, partial colocalization of ERdj8 with PIS may imply that ERdj8 affects the supply of phospholipids to the autophagosomes at the ER subdomain. VMP1 also colocalizes with PIS and CEPT1 on the ER subdomain, which suggests a functional relationship in autophagosome formation.

## Conclusions

In this review, we have discussed how the ER is related to autophagosome formation. In this regard, current studies are expected to shed light on the mechanisms that regulate the size of the autophagosomes.

## Data Availability

Not applicable.
